# Insights into transmission dynamics of *Mycobacterium tuberculosis* complex in Nepal

**DOI:** 10.1186/s41182-022-00400-z

**Published:** 2022-01-11

**Authors:** Yogendra Shah, Sarad Paudel, Kishor Pandey, Govind Prasad Gupta, Eddie Samuneti Solo, Jagadish Joshi, Dhan Kumar Pant, Basu Dev Pandey

**Affiliations:** 1 COVID-19 PCR Laboratory, Seti Provincial Hospital, Dhangadhi, Kailali Nepal; 2 Pathobiology and Diagnostic Investigation Department, College of Veterinary Medicine, Michigan State University, East Lansing, MI USA; 3 Central Department of Zoology, Tribhuvan University, Kirtipur, Kathmandu, Nepal; 4 Everest International Clinic and Research Center, Kalanki, Kathmandu, Nepal; 5 Department of Clinical Microbiology, School of Physiotherapy and Paramedical Science, Lovely Professional University, Phagwara, India; 6 Department of Pathology and Microbiology, University Teaching Hospital, RW 1X, Ministry of Health, Lusaka, Zambia; 7 Department of Emergency Medicine, Seti Provincial Hospital, Dhangadhi, Kailali Nepal; 8 National One Health Alliance for Nepal, Tahachal, Kathmandu, Nepal; 9 Department of Molecular Epidemiology, Institute of Tropical Medicine (NEKKEN), Nagasaki University, Nagasaki, Japan

**Keywords:** Tuberculosis, Zoonotic tuberculosis, Multi-drug resistance, One health, Nepal

## Abstract

Tuberculosis (TB) is an infectious disease caused by *Mycobacterium tuberculosis* complex (MTBC) in humans and animals. Numbers of multi drug resistance TB (MDR-TB), extrapulmonary TB (EPTB) and zoonotic TB cases are increasingly being reported every year in Nepal posing a major public health problem. Therefore, the Government of Nepal should act immediately to strengthen the screening facilities across the country to be able to identify and treat the TB infected patients as well as detect zoonotic TB in animal species. Endorsement of One Health Act by the Government of Nepal is an opportunity to initiate the joint programs for TB surveillance among human and animal species using one health approach to reduce the TB burden in Nepal.

## Dear Editor,

Tuberculosis (TB) is an air borne infectious disease caused by MTBC in human and animal species and poses a major public health problem particularly pulmonary TB. Extra-pulmonary TB (EPTB) is also involved and contributes 15–30% of cases [1]. TB has been described to infect one third of the world’s population [2]. According to WHO, it is estimated that 10 million people fell ill and 1.4 million deaths occurred due to TB worldwide in 2019 [2]. Majority of deaths caused by TB were in developing countries with more than half cases occurring in Asia and Africa [2]. Multi-drug resistant tuberculosis (MDR-TB), resistant to rifampicin and isoniazid, is an emerging threat for successful TB control in the Indian subcontinent including Nepal [2–5].

Nepal is one of the South Asian countries, which reported large number of TB cases with estimated prevalence of 416 and incidence of 245 per 100,000 populations in 2018 [2]. National TB prevalence survey report showed that TB burden in Nepal is about 117,000 (88,000–145,000) people with TB disease are living in Nepal. Among those, 69,000 (41,000–103,000) people developed TB in 2018. This figure is much higher (1.6 times) than previously estimated [1, 2, 6]. Therefore, Nepal needs an urgent action plan to accelerate TB response to end TB by 2035 as a target goal set by WHO.

In Nepal, TB caused by different members of MTBC was detected in different animal species including Asian elephants, greater-one-horned rhinoceros, deer species and blue bull by employing molecular genotyping tools among different particular host [7–10]. Furthermore, two captive Asian elephants were reportedly infected with mixed MTB lineages (Indo-oceanic and Beijing/CAS-Delhi lineages) in Nepal (Table 1), by employing whole genomic sequencing analysis [11]. Similarly, TB screening carried out using single intradermal tuberculin test in cattle/buffaloes in 60 households of TB infected patients showed the higher risk of bovine TB transmission from animal to human or vice versa [12]. Likewise, a recent study in 123 cattle on bovine TB using different tests revealed that 12 (9.76%) were reactive on the comparative tuberculin test; 46 (37.4%) were positive on rapid test and 7 (5.7%) were positive on ELISA method which shows the high risk of transmission of zoonotic TB i.e. bovine TB at cattle-human interface in Nepal [13]. These studies show that there might be interspecies transmission of TB among human and animal species in Nepal.

**Table 1:** TB diagnosis in different animal species in Nepal

Year	Location (Nepal)	Sample type	Sample size	Animal species	Necropsy	Phenotypic	Genotyping tools	*Mycobacterium* Spp.	References
2007–2010	Chitwan National Park, Koshi Tappu Wildlife Reserve	Lung tissues	3	Asian Elephant	Granulomatous nodules in the lungs and bronchial lymph nodes with caseous foci	Culture in L-J, Phenotypic drug susceptibility testing	Multiplex PCR, Spoligotyping, MLVA, SNP analysis, Drug resistance gene sequencing	*M. tuberculosis*	Paudel et al. [7]
2014	Captive Wild animal facility	Lung and extra pulmonary granulomatous lesions	2	Deer and Blue bull	No details	Microscopy Culture DNA extraction	Spoligotyping MIRU-VNTR MLST	*M. orygis*	Thapa et al. [8]
2015	Chitwan National Park	Lung tissues	1	Greater-one-horned rhinoceros	Several granulomatous lesion observed in lungs with encapsulated and contained caseous necrotic material	Culture in L-J, Phenotypic drug susceptibility testing	Spoligotyping, MIRU-VNTR, Multiplex PCR, Region of difference MLST	*M. orygis*	Thapa et al. [9]
2013	Chitwan National Park	Lung Tissues	2	Asian elephant	Tuberculous lesions in lungs	Culture in L-J, Phenotypic drug susceptibility testing	Spoligotyping, LSP, Drug resistance gene sequencing	*M. tuberculosis mixed infection* Indo-Oceanic, East African– Indian (CAS-Delhi) lineages; Indo-Oceanic and East Asian (Beijing) lineages	Paudel et al. [10]

MLVA, Multi-locus variable number of tandem repeat analysis; LSP, Large Sequence Polymorphisms

According to National Tuberculosis programme (NTP), drug resistance survey carried out from 2011 to 2012 revealed that the burden of drug resistant forms of TB was increasing as 9.3% of new patients were found resistant to at least one anti-tuberculosis drug. Due to expansions in diagnostic services of TB; case finding rates among new case has remarkably increased in recent years. For instance, new MDR-TB contribution has increased drastically in the last 5 years (14.6% in 2015/2016, 15.3% in 2016/2017, 18.3 in 2017/2018, 32% in 2018/2019 and 59% in 2019/2020) [14]. Similarly, EPTB status in Nepal has shown increasing trends in the last 4 years (28.94% in 2017/18, 29% in 2017/2018; 29% in 2018/2019, 32% in 2019/2020) [14]. Furthermore, a research that was conducted in different tertiary care centers showed that 26.79% and 69.10% patients had EPTB infection in various sites [15, 16]. Conclusively, the numbers of MDR-TB, EPTB and zoonotic TB cases are increasing in Nepal and the reasons behind the increasing trends are still unknown. Therefore, comprehensive studies (employing whole-genome sequencing) on MDR-TB, EPTB and zoonotic TB in Nepal are needed to better understand their transmission, particularly the zoonotic TB interface between wild and domestic animals.

In Nepal, One Health (OH) approach has been endorsed by the Government of Nepal (GoN) in 2019 through the donor funded projects together with inter-governmental agencies i.e. World Health Organization, Food and Agricultural Organization and Animal Health as well as with close coordination with International & National non-governmental organization. The OH act in Nepal with OH approach has been successfully executed for example in tackling against the antimicrobial resistant problem in Nepal by Fleming fund country grant, control of rabies virus and Japanese encephalitis virus in close coordination and collaboration among concerned agencies [17, 18].

Therefore, the NTP in Nepal should be strengthened to international standards with availability of general laboratory services including molecular rapid and low cost assays like loop mediated isothermal amplification (LAMP) that can be used for early detection and surveillance of bovine TB in animals and humans in resource-limited countries like Nepal [19]. Surveillance system should be strengthened to monitor the MTBC transmission in Nepal by providing better TB-screening and effective treatment and diagnosis strategies within the country and in border areas. Nepal shares a long border with India, a country with the highest TB burden in the world, therefore collaboration between public and private sectors is required. Furthermore, the GoN should act immediately to strengthen the screening facilities across the country to identify and treat the TB infected animal species. The recent international recommendations of OH strategy should be leveraged to reduce the TB threat in Nepal.

## Data Availability

The information on TB diagnosis in different animal species in Nepal available from references [7–10]

## Abbreviations

TB: Tuberculosis
MTBC: *Mycobacterium tuberculosis* Complex
MDR-TB: Multi drug resistant TB
EPTB: Extrapulmonary TB
CAS: Central Asian Strain
AFB: Acid fast bacilli
GON: Government of Nepal
NTP: National Tuberculosis programme
OH: One Health
